# Human CD33 deficiency is associated with mild alteration of circulating white blood cell counts

**DOI:** 10.1371/journal.pgen.1011600

**Published:** 2025-03-05

**Authors:** John Dominy, Jirong Bai, Christopher Koch, Maleeha Zaman Khan, Shareef Khalid, Jonathan H. Chung, Madhura Panditrao, Lulu Liu, Qi Zhang, Muhammad Jahanzaib, Muhammad Rehan Mian, Muhammad Bilal Liaqat, Syed Shahzaib Raza, Riffat Sultana, Anjum Jalal, Muhammad Hamid Saeed, Shahid Abbas, Fazal Rehman Memon, Mohammad Ishaq, Kashif Saleheen, Asif Rasheed, Allan Gurtan, Danish Saleheen

**Affiliations:** 1 Biomedical Research at Novartis, Cambridge, Massachusetts, United States of America; 2 Center for Non-Communicable Diseases, Karachi, Sindh, Pakistan; 3 Department of Medicine, Columbia University Irving Medical Center, New York, New York, United States of America; 4 Karachi Institute of Heart Diseases, Karachi, Sindh, Pakistan; 5 Punjab Institute of Cardiology, Lahore, Punjab, Pakistan; 6 Faisalabad Institute of Cardiology, Faisalabad, Punjab, Pakistan; 7 Red Crescent Institute of Cardiology, Hyderabad, Sindh, Pakistan; 8 Department of Cardiology, Columbia University Irving Medical Center, New York, New York, United States of America; HudsonAlpha Institute for Biotechnology, UNITED STATES OF AMERICA

## Abstract

The single pass transmembrane protein CD33 is enriched in phagocytic and hematopoietic cell types, such as monocytes. CD33 is thought to be associated with immune cell function, susceptibility to Alzheimer’s disease, and rare leukemias. Antagonism or genetic ablation of *CD33* has been proposed to treat Alzheimer’s disease, hematological cancers, and as a selection mechanism for enriching genetically altered blood cells. To understand the impact of chronic CD33 loss or ablation, we describe individuals who we confirmed to be missing *CD33* due to germline loss of function variants. Through PheWAS-based approaches using existing whole exome biobanks and bespoke phenotyping using recall-by-genotype (RBG) studies, we show that *CD33* loss of function alters circulating white blood cell counts and distributions, albeit mildly and with no overt clinical pathology. These findings indicate that chronic CD33 antagonism/ablation is likely to be safe in humans.

## Author summary

CD33 is expressed on certain cell types in the human immune system. Its exact role in human health is not clear but it may be linked to Alzheimer’s disease and leukemias. Therapies are being designed to reduce or eliminate CD33 function to treat disease in humans, but the consequences of long term loss of CD33 in humans are uncertain. To help derisk future CD33 lowering therapies, we identified individuals who have been missing either one or two copies of functional CD33 since birth. Loss of CD33 was associated with mild changes in blood cell counts but otherwise no obvious clinical presentations. These findings suggest, but do not conclusively prove, that elimination of CD33 does not lead to severe health issues or chronic infection.

## Introduction

CD33 molecule (CD33) is a Type 1 single pass transmembrane protein that belongs to the Siglec (Sialic acid-binding immunoglobulin-type lectins) protein family. This class of protein is broadly characterized by the presence of an N-terminal V-type immunoglobulin domain and the ability to bind sialylated glycan ligands. CD33 may preferentially bind α-2,6-linked sialic acid glycans [1].

CD33 expression is enriched in multiple phagocytic and hematopoietic cell types of mammals, including macrophages, monocytes, microglia, dendritic cells, hematopoietic progenitors, some lymphoid cell types, and myelomonocytic precursor types [1,2]. This enrichment of expression suggests a role in immune system function, consistent with the broader function of Siglec family members in regulating inhibition of immune cell activity. Siglecs possess a cytoplasmic immunoreceptor tyrosine-based inhibition motif (ITIM). Upon ligand binding by the Siglec, the ITIM is phosphorylated and recruits SH2-domain proteins that reduce immune cell activation. For example, Siglec protein CD22 negatively regulates B cell receptor activity, as demonstrated by B cell hyperactivation after challenge with a T cell dependent antigen in *Cd22* knockout mice [3].

CD33’s exact function in humans remains to be ascertained. Gleaning insights into CD33 function from mouse has been confounded by the significant protein sequence divergence from human (~50% sequence identity) and the absence, in mouse, of a competent ITIM sequence. *Cd33* knockout mice do not show any immune phenotype [4], whereas *in vitro* studies with human CD33 suggest a role for CD33 in inhibiting immune cell activity. Human monocytes treated with either a CD33 antibody or a CD33 siRNA exhibited elevated cytokine production [5]. Genome and exome wide association studies have shown non-coding variants in the *CD33* locus to be associated with changes in white blood cell counts, also suggesting that this protein plays a role in immune cell regulation [6–8]. Multiple genome wide association studies (GWAS) have identified single nucleotide polymorphisms (SNPs) in *CD33* that are associated with a change in the risk for Alzheimer’s disease or late onset dementia [6–9].

**Fig 1:**
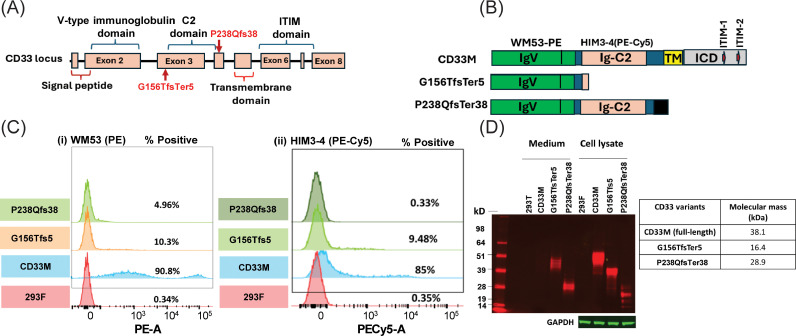
In vitro characterization of CD33 variants. (A) A schematic of the human CD33 gene locus. Exons are overlaid with the protein domains that they correspondingly encode. Relative positions of the two most abundant frameshift variants from PGR are indicated in red. (B) Synthetic CD33 gene constructs for cell surface expression assays. The relative locations of binding epitopes for anti-CD33 monoclonal antibodies WM53 and HIM3-4 are highlighted. The full-length CD33M polypeptide consisted of the extracellular region, containing Ig-V and Ig-C2 domains, the transmembrane domain (TM), and intracellular domain (ICD). ICD contains two immunoreceptor tyrosine-based inhibitory motifs (ITIM-1 and ITIM-2). (C) *FACS assessment of CD33 variant expression. CD33M is highly expressed on the surface of transfected 293F cells but TM-free truncation variants G156TFsTer5 and P238QFsTer38 are nominally expressed, as assessed by (i) cells stained with Ig-V domain-targeting monoclonal antibody WM53-*PE or (ii) Ig-C2 domain-targeting monoclonal antibody HIM-3-4 (PE-Cy5). N = 3 independent assays. (D) Immunoblot *assays for the expression of CD33M and truncation mutants. Medium and lysates samples were derived from 293T cultures 72 hours post transfection. Protein expression was probed with rabbit anti-human CD33 Ab134115 in combination with goat anti-rabbit IgG Dylight 680. CD33M medium showed no secreted extracellular domain polypeptide fragment. Secreted G156TfsTer5 migrated at a higher molecular weight than predicted, presumably due to glycosylation; P238QfsTer38 migrated closer to its expected molecular weight. CD33M was expressed in the cell lysate at levels higher than the truncation variants. Again, cell associated G156Tfs5Ter5 migrated at a higher-than-expected molecular weight. P238QfsTer38 exhibited evidence of degradation.*

Identification and characterization of individuals with biallelic germline loss of CD33 would further the understanding CD33’s pathophysiological function in humans. To date, only isolated singleton individuals with very low or absent CD33 expression and hematological cancer have been reported but the small number of cases precludes any generalization of potential biology [10]. Public human genetic repositories, such as the Genome Aggregation Database (gnomAD version 4.1), indicate the existence of germline homozygous carriers of predicted loss of function (pLOF) variant NP_001763.3:p.G156TfsTer5, present at an allele frequency of ~2.4% in global populations (see Fig 1A for *CD33* locus schematic). Although predicted LOF variant carriers exist, there are no reports experimentally confirming these variants to be LOF or characterizing their direct phenotypic impact in humans. Understanding the phenotypic impact of CD33 LOF may inform on-going industry efforts to antagonize or genetically delete CD33 for Alzheimer’s disease [11] as well as various cell therapies for the treatment of hematological cancers in which CD33 is either genetically deleted as a mechanism to improve therapeutic cell selection and engraftment or CD33 itself is an effector target of the cell therapy [12–15].

To understand CD33 function in humans, we employed the Pakistan Genome Resource (PGR), which is the world’s largest biobank of human homozygous pLOF carriers (knockouts) identified through whole-exome sequencing of >80,000 participants [16]. With the aid of PGR and its associated infrastructure, we identified homozygous carriers of *CD33* pLOF variants, confirmed experimentally *in vitro* that these variants are loss of function, and evaluated whether CD33 LOF is associated with any overt clinical phenotypes. We complemented these efforts with an analysis of CD33 pLOF variants in UKB and their effect on blood cell counts and other phenotypes.

## Results

### Identification of CD33 frameshift variants in PGR and their biochemical characterization

A survey of the PGR (see methods) identified 19 pLOF variants that included protein truncating variants, frameshift variants, and splice disruptors (Table 1). Among these variants, the two most common were NP_001763.3:p.G156TfsTer5 and NP_001763.3:p.P238QfsTer38, with alternate allele frequencies of 0.12% and 0.039%, respectively. These two variants were selected for further *in vitro* characterization to evaluate impact of the frameshift variant on expression. The predicted termination of these two proteins occurs after the IgV domain but before the transmembrane domain of canonical reference CD33 protein sequence and there is thus a theoretical possibility that they could result in soluble, secreted proteins (Fig 1A).

**Table 1:** List of high confidence pLOF variants in PGR.

Chr:Pos:Ref:Alt (GRCh38)	Variant effect	Predicted coding consequence	Heterozygous carriers	Homozygous carriers
19:51225121:GC:G	Frameshift	p.Pro2ArgfsTer28	1	0
19:51225139:GC:G	Frameshift	p.Leu9CysfsTer21	1	0
19:51225157:T:G	Splice donor		1	0
19:51225217:G:A	Splice acceptor		7	0
19:51225249:G:GC	Frameshift	p.Gln24ProfsTer75	1	0
19:51225357:CTGGT:C	Frameshift	p.Trp60SerfsTer18	1	0
19:51225359:G:A	Stop gained	p.Trp60Ter	1	0
19:51225445:A:T	Stop gained	p.Arg89Ter	2	0
19:51225528:C:G	Stop gained	p.Tyr116Ter	1	0
19:51225847:CCCGG:C^ǂ^	Frameshift	p.Gly156ThrfsTer5	195	4
19:51225920:G:A	Stop gained	p.Trp179Ter	3	0
19:51225982:AC:A	Frameshift	p.Pro201HisfsTer10	5	0
19:51226021:CA:C	Frameshift	p.Gln213ArgfsTer2	12	0
19:51226321: ACCCAACAACTGGTATCTTT:A^ǂ^	Frameshift	p.Pro238GlnfsTer38	64	1
19:51226358:T:G	Splice donor		1	0
19:51235245:CT:C	Frameshift	p.Phe279SerfsTer3	2	0
19:51235678:T:C	Splice donor		3	0
19:51239634:GA:G	Frameshift		2	0
19:51239663:AAT:A	Frameshift		1	0

Chr, chromosome; pos, position; Ref, reference allele; Alt, alternate allele. Predicted coding consequence is relative to CD33 isoform 1, NP_001763.3.

ǂ -2 participants were compound heterozygotes for these two variants and were thus considered as a KO for the analysis.

Mammalian expression constructs corresponding to the predicted cDNAs of reference allele (CD33M) and frameshift variant-containing human *CD33* were generated to test their levels of surface expression and potential secretion (Fig 1B). Transient transfection of HEK293F cells with reference *CD33* resulted in significant levels of surface protein expression by FACS when using an IgV domain targeting antibody, WM53, or an Ig-C2 domain targeting antibody, HIM-3-4 (Fig 1C). By contrast, neither p.G156TfsTer5 nor p.P238QfsTer38 showed significant levels of surface expression when evaluated under the same conditions. Western blots of transiently transfected HEK293F cell lysates and medium were conducted to ascertain total expression of the variants from cDNA cassettes as well as their potential secretion into the media. Probing of total cell lysates demonstrated expression of reference CD33M as well as the frameshift variants, indicating that cells could support generation of the frameshift variants (Fig 1D). Evaluation of CD33 signal in the media revealed detectable levels of secreted frameshift variants but not CD33M reference allele. In aggregate, these data indicated that frameshift variant proteins were efficiently produced by cells and, if the frameshift variant transcripts were able to escape nonsense mediated mRNA decay in human carriers, a secreted protein could be generated. Measurement of CD33 protein levels in both sera and cells of human variant carriers was therefore necessary to resolve this.

### Recall-by-genotype of CD33 variant carriers from the Pakistan Genomic Resource

The population of Pakistan has a high rate of consanguinity, which can result in homozygous enrichment of otherwise rare variants, including pLOF variants [16]. The Pakistan Genomic Resource (PGR) at the Center for Non-Communicable Diseases in Karachi, Pakistan contains a large biobank of individuals (> 250,000) who have consented to participate in sequencing and recontact studies. With the aid of the whole exome sequencing data from the PGR, carriers of the two most common frameshift variants, p.G156TfsTer5 and p.P238QfsTer38, as well as a less common frameshift variant, p.Q213RfsTer2, were recontacted in a recall-by-genotype (RBG) study. These individuals, along with recruited consenting family members, were (1) genotyped at CD33 by Sanger sequencing to confirm carrier status and zygosity, and (2) phenotypically characterized for a wide array of traits including anthropometry, blood pressure, a focused lipid panel, complete blood counts, and personal and family medical histories.

**Fig 2:**
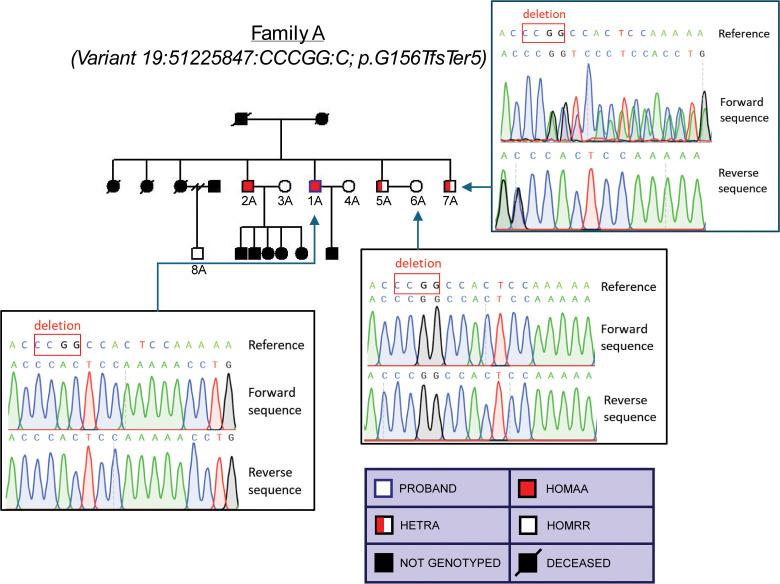
Pedigree of homozygous p.G156TfsTer5 variant carrier family A.

**Fig 3:**
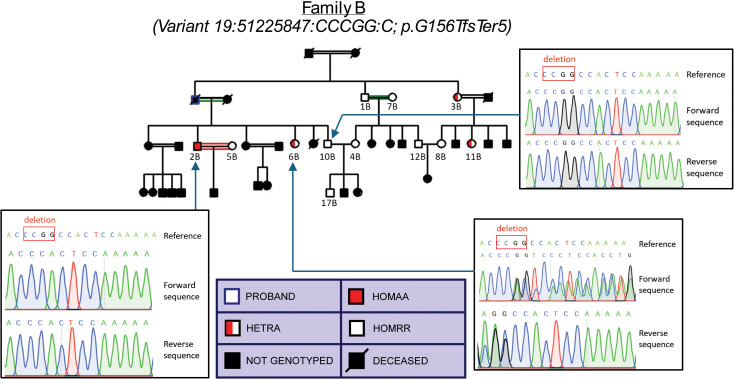
Pedigree of homozygous p.G156TfsTer5 variant carrier family B.

Through RBG of 14 probands, 114 individuals were identified across 14 different families: 11 families for the variant p.G156TfsTer5, 2 families for the variant p.P238QfsTer38, and 1 family for the variant p.Q213RfsTer2. A breakdown of the variant carrier numbers, as ascertained by Sanger sequencing, is shown in Table 2. Importantly, the recall studies successfully identified 3 homozygous p.G156TfsTer5 variant carriers: 2 brothers, ages 55 (participant ID 1A) and 58 (participant ID 2A), in family A (Fig 2) and a 35 year-old male (participant ID 2B) in family B (Fig 3).

**Table 2:** A breakdown of CD33 frameshift variant carriers identified through recall by genotype studies.

Variant	Noncarriers	Heterozygous carriers	Homozygous carriers	gnomAD overall frequency	gnomAD South Asian frequency	CADD Score	ClinVar Classification
p.G156TfsTer5	62	30	3	0.023707	0.000824	24.1	Benign
p.Q213RfsTer2	6	2	0	0.000003	0.000055	19.5	n/a
p.P238QfsTer38	6	5	0	0.000682	0.000176	22.8	Likely Benign

**Fig 4:**
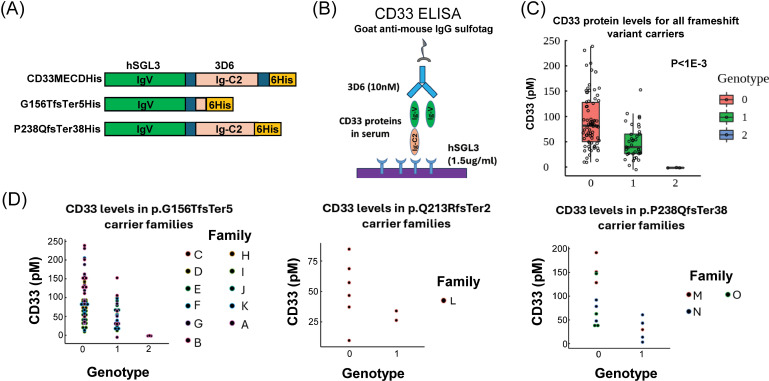
Summary of participant serum CD33 concentration assays. (a) Recombinant CD33 protein constructs used to evaluate CD33 detection assays. hSGL3, anti-CD33 human IgG1 binds to an epitope located at the N-terminus of Ig-V domain. 3D6, mouse anti-human CD33 mAb binds to the Ig-C2 region. (b) Schematic of quantitative binding assay for serum CD33 levels. (c) CD33 serum concentrations across all frameshift variant genotypes, 0 = noncarrier, 1= heterozygous variant carrier, 2= homozygous variant carrier. CD33 serum levels among the three genotype groups were statistically significant (P<1E-4). (D) CD33 levels for each frameshift variant stratified by genotype and family ID.

**Fig 5:**
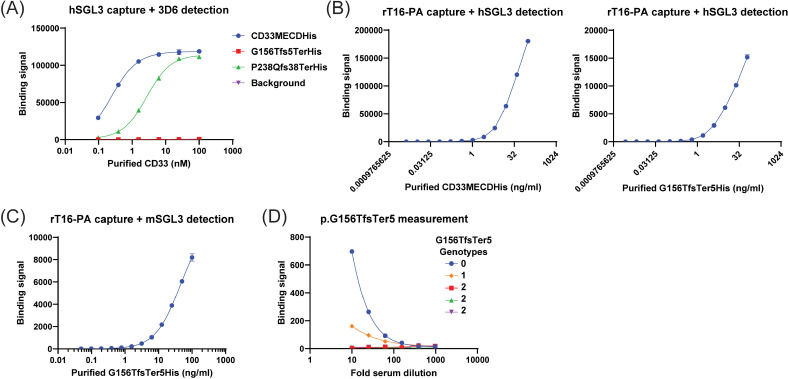
Independent binding assay confirms that circulating levels of CD33 protein in G156TfsTer5 homozygous carriers are below detection level. (A) G156TfsTerHis, which is missing most of the Ig-C2 domain that is recognized by 3D6 antibody, is poorly detected in the ELISA format shown in Fig 4B. (B) An ELISA assay designed with rabbit anti-human CD33 polyclonal antibody rT16-PA (Cat# 12238-T16, SinoBiological; immunogen = M1-H259 of CD33) to capture CD33 polypeptides followed by detection with hSGL3 antibody can directly detect G156TfsTer5. Signals from purified, recombinant CD33MECDHis and G156TfsTer5His tag constructs are shown. (C) Reformatting of hSGL3 to a murine constant region (mSGL3) preserves detection of G156TfsTer5 protein. (D). *Measurement of G156TFsTer5 levels in select participant sera samples from the recall by genotype study. Homozygous reference:* “*0” genotype; Heterozygous carrier:*“*1” genotype; Homozygous G156TfsTer5 carrier:* “*2” genotype.*

To ascertain whether the frameshift variants resulted in any secreted CD33 isoforms in the circulation of variant carriers, two sandwich-format MesoScaleDiscovery (MSD) assays for the CD33 ectodomain were established In the first assay, monoclonal antibody hSGL3 recognizing the common IgV domain of all isoforms was used to capture CD33 protein from sera followed by detection with a monoclonal antibody, 3D6, that binds to the Ig-C2 domain (Fig 4A and 4B). Soluble CD33 protein was detectable in the sera of homozygous reference carriers (Fig 4C, red bar) and levels of free CD33 protein dropped off in a significant, gene-dosage dependent manner for the frameshift variant carriers (Fig 4C, blue and green bars). Homozygous variant carriers had non-detectable levels of protein in circulation. This general pattern was seen across all of the frameshift variants and in all families (Fig 4D). During assay development, it was observed that capture and detection of recombinant purified G156TfsTer5 with the hSGL3/3D6 antibody pair was poor (Fig 5A), perhaps due to reduced ability of this variant to bind to 3D6. As a consequence, a second assay using rabbit polyclonal antibody rT16-PA to capture CD33 protein and a murine version of hSGL3 to detect G156TfsTer5 was developed (Fig 5B and 5C). hSGL3 was reformatted to a murine isotype to reduce background. A subset of recall by genotype sera samples, including all 3 homozygous G156TfsTer5 samples, were tested in this assay (Fig 5D). Total binding signal in this assay format reduced in a gene dosage-dependent manner with homozygous G156TfsTer5 carriers showing no detectable levels of CD33 protein in sera even at the lowest sample dilution (1:10). These data indicated that, in contrast to the secreted proteins generated by the frameshift variants *in vitro*, there was no secreted protein produced *in vivo*, possibly due to nonsense mediated decay of the variant transcripts.

**Fig 6:**
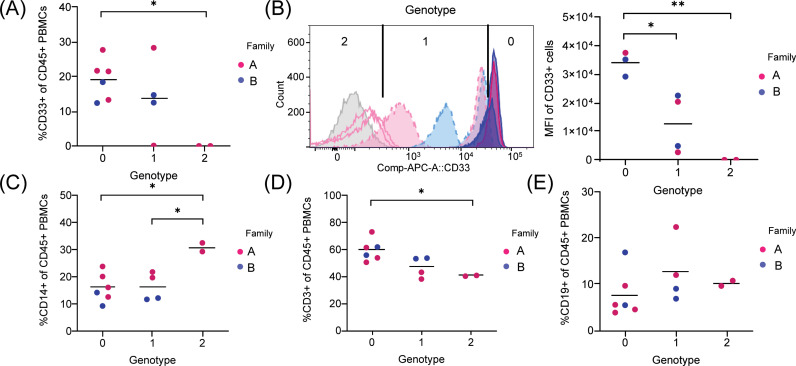
FACS-based evaluation of CD33 levels and multi-lineages from PBMCs in families A and B. (A) CD33+ myeloid cells as a percentage of total CD45+ cells. (B) Comparison of levels of surface CD33 expression in p.G156TfsTer5 carrier families. Histograms of CD33-APC of individual participates are shown in left and statistical analysis of mean fluorescence intensity (MFI) is shown in right. Histograms are colored by genotype. Unstained control cells are shown in grey. (C) CD14^+^ *monocytes as a percentage of total CD45*^+^ *cells*. (D) *CD3*^+^ *T cells as a percentage of total CD45*^+^ *cells*. (E) *CD19*^+^ *B cells as a percentage of total CD45*^+^ cells. *P <5E-2, **P *<1E-2 compared between indicated groups.* P *values were determined by one-way analysis of variance (ANOVA).*

To confirm that CD33 expression was affected at the surface of relevant immune cell types, peripheral blood mononuclear cells (PBMCs) were isolated from participants in families A and B, which contained heterozygous and homozygous carriers of the p.G156TfsTer5 variant as well as homozygous reference allele carriers (see Figs 2 and 3 for pedigrees comprising these individuals along with sample Sanger sequencing results). Cell surface expression of CD33 protein was evaluated in the PBMCs by FACS using a monoclonal antibody (clone P67.6) that recognizes the common IgV domain of all CD33 isoforms [17]. Surface expression of CD33 on CD45^+^ cells was clearly detectable in homozygous reference carriers (Genotype 0, Fig 6A). This expression was ablated in PBMCs of homozygous p.G156TfsTer5 variant carriers (Genotype 2, Fig 6A). Heterozygous variant carriers (Genotype 1) had intermediate percentages of CD33^+^ expression that varied between those found in homozygous reference carries to complete loss of expression (Fig 6A). Quantification of surface CD33 expression by mean fluorescence intensity (MFI) showed a significant gene dosage-dependent decrease in expression (Fig 6B).

Although all myeloid cells of homozygous variant carriers showed no detectable expression of CD33, the monocytes in the PBMCs of these carriers still expressed CD14 (Genotype 2, Fig 6C). Moreover, compared to homozygous and heterozygous reference carriers, homozygous variant carriers displayed slight but significantly higher frequency of CD14^+^ monocytes (Fig 6C). Correspondingly, the percentage of peripheral CD3^+^ T cells was slightly lower in homozygous variant carriers (Genotype 2, Fig 6D). By contrast, CD19^+^ B cells as a percentage of total CD45^+^ hematopoietic cells in circulation were equivalent across all family members regardless of carrier status (Fig 6E). Although the number of participants for which PBMCs could be profiled was limited, the data show that loss of CD33 was resulting in a subtle yet significant change in the immune cell compartment.

To determine whether CD33 LOF was resulting in any other phenotypic changes across all participants in the RBG, 47 phenotypes were analyzed for potential associations with CD33 LOF carrier status (Table 3). No phenotypes were found to be significantly associated with CD33 LOF after multiple testing correction (P = 1.1E-3). This includes complete blood cell count with differentials, suggesting that the loss of CD33 did not produce a large global perturbation in immune cell distributions—consistent with the findings from the PBMC analyses of a subset of variant carriers. Several nominal and weak associations were found: higher waist-to-hip ratio (P = 4.6E-2), higher cholesterol (P = 1.3E-2), higher triglycerides (P = 3.4E-2), higher LDL cholesterol (LDL-C) (P = 1.3E-2), and higher rate of myocardial infarction (MI, P = 1E-2) and cataracts (P =4.4E-2). The observation of higher incidence of MI may be driven by ascertainment bias since a subset of CD33 LOF carrier probands were recruited from an MI case cohort within PGR. The identification of three adult homozygous LOF males who have all had children also indicates that complete LOF of CD33 does not result in male sterility.

**Table 3:** Distribution of baseline characteristics of recall-by-genotype participants per genotype.

		HomRR	HetRA	HomAA	
	Count	Median (IQR) or %	Median (IQR) or %	Median (IQR) or %	P value (additive)*
**Quantitative Traits**					
Age	74:37:3	35 (23,48)	40 (28,57)	55 (45,57)	
Gender (Female)	50:12:0	67.6%	32.4%	0%	
BMI (kg/m^2)	72:34:3	24 (20,3020,30)	23.2 (20.6,27.8)	27.2 (23.8,29.4)	6.1E-1
Height (cm)	73:35:3	156 (152,162)	163 (156.5,168)	166 (164,166.5)	8.3E-1
Weight (kg)	72:34:3	60.45 (51.17,72.9)	63.35 (54.5,74.35)	75.8 (66,79.4)	5.8E-1
WHR	72:36:3	0.87 (0.82,0.92)	0.93 (0.88,0.96)	0.90 (0.88,0.91)	4.6E-2
Waist (cm)	72:36:3	86 (72,97)	87 (76,98)	94 (89,95)	2.1E-1
Hip (cm)	72:36:3	96 (87,106)	93.5 (85,103)	107 (99,108)	6.5E-1
BP systolic (mmHg)	72:36:3	125 (113,139)	129 (121,145)	139 (127,142)	6.7E-1
BP diastolic (mmHg)	72:36:3	82 (75,90)	85 (78,93)	89 (82,91)	4.9E-1
Heart rate (bpm)	64:34:3	87 (78,94)	88 (80,94)	94 (88,95)	4.8E-1
Fat mass (kg)	64:32:3	19 (11,2711,27)	13 (8,208,20)	17 (15,2115,21)	6.1E-1
Fat free mass (kg)	64:32:3	43 (39,48)	47 (41,53)	58 (50,58)	6.9E-1
Cholesterol (mg/dl)	74:37:3	148.8 (126.6,182.9)	163.8 (139.6, 215.2)	176.7 (173.8,192.6)	1.3E-2
Triglycerides (mg/dl)	72:37:3	145.4 (87.5,240.4)	181.2 (110.7,266.9)	244.6 (237.8,256.9)	3.4E-2
HDL-C (mg/dl)	74:37:3	36 (28,41)	34 (29,40)	31 (30,3230,32)	8.9E-1
LDL-C (mg/dl)	73:36:3	83 (61,102)	96 (72,130)	95 (94,111)	1.3E-2
Creatinine (mg/dl)	73:37:3	0.64 (0.51,0.76)	0.73 (0.64,0.91)	0.78 (0.73,0.86)	8.3E-1
eGFR (mL/min/1.73m^2)	73:37:3	118 (100,154)	101 (87,132)	102 (85,113)	7.31E-1
HbA1c (%)	65:30:2	5.4 (5.0,5.8)	5.6 (5.2,6.0)	8.7 (7.8,9.7)	1.7E-1
Glucose (mg/dl)	74:37:3	86 (75,103)	88 (79,110)	158 (137,261)	6.5E-1
AST (U/L)	74:37:3	21 (18,2618,26)	22 (18,2718,27)	22 (22,3022,30)	4.1E-1
ALT (U/L)	74:37:3	15 (13,2513,25)	22 (13,3113,31)	27 (26,3526,35)	6.8E-1
White Blood Cells (10^9/ L)	58:29:2	6.1 (4.6,7.3)	6.4 (5.8,7.8)	4.8 (4.3,5.4)	6.5E-1
Red Blood Cells (10^12/ L)	58:29:2	4.9 (4.4,5.5)	5.0 (4.6,5.5)	4.7 (4.6,4.8)	8.8E-1
Hemoglobin (g/dl)	58:29:2	13.6 (12.0,15.2)	14.7 (12.5,15.7)	13.2 (12.9,13.4)	4.5E-1
Hematocrit (%)	58:29:2	41.5 (37.5,46.2)	43.4 (37.9,48.1)	40.0 (39.2,40.7)	3.7E-1
MCV (fl)	58:29:2	86 (80,89)	84 (82,91)	85 (85,85)	3.9E-1
MCH (pg)	58:29:2	28 (26,3026,30)	28 (27,3027,30)	28 (28,2828)	3.4E-1
MCHC (g/dl)	58:29:2	32.4 (31.5,33.3)	33.2 (32.3,33.7)	32.9 (32.9,33.0)	7.0E-1
Platelets (10^9/ L)	58:29:2	230 (150,270)	231 (175,273)	168 (157,179)	5.3E-2
Neutrophils (%)	58:29:2	52.0 (39.5,58.4)	53.8 (48.7,60.8)	54.7 (54.5,54.9)	4.8E-1
Lymphocytes (%)	58:29:2	35.0 (29.2,46.5)	30.4 (27.4,36.5)	24.2 (24.1,24.4)	7.5E-1
Monocytes (%)	58:29:2	8.0 (6.4,9.6)	7.7 (6.8,8.9)	13.5 (12.4,14.5)	7.0E-1
Eosinophils (%)	58:29:2	2.6 (1.5,3.9)	3.1 (2.4,4.6)	4.6 (3.5,5.7)	2.7E-1
Basophils (%)	58:29:2	1.8 (0.9,3.9)	1.7 (0.9,1.9)	3.1 (2.6,3.5)	6.7E-1
Urinary microalbumin (mg/dl)	69:35:3	0.52 (0.20,2.01)	0.54 (0.19,1.64)	0.47 (0.44,2.90)	5.5E-1
**Clinical Outcomes**					
Myocardial Infarction	1:9:1 Cases 73:28:2 Controls	1.4%	24.3%	33.3%	1.0E-2
Dyslipidemia	1:5:0 Cases 61:26:3 Controls	1.6%	16.1%	0.0%	1.8E-2
Cataract	1:4:0 Cases 61:27:2 Controls	1.6%	12.9%	0.0%	4.4E-2
Hypertension	19:15:1 Cases 55:22:2 Controls	25.7%	40.5%	33.3%	2.5E-1
Tuberculosis	3:4:0 Cases 71:33:3 Controls	4.1%	10.8%	0.0%	1.4E-1
Diabetes Mellitus	12:6:2 Cases 62:31:1 Controls	16.2%	16.2%	66.7%	7.3E-1
Rheumatoid Arthritis	5:2:0 Cases 57:29:2 Controls	8.1%	6.5%	0.0%	4.6E-1
Recurrent Chest Infections	4:2:0 Cases 70:35:3 Controls	5.4%	5.4%	0.0%	2.4E-1
Allergies	11:7:0 Cases 63:30:3 Controls	14.9%	18.9%	0.0%	9.3E-1
Hepatitis	3:2:0 Cases 71:35:3 Controls	4.1%	5.4%	0.0%	8.9E-1
Chronic Kidney Disease	3:2:0 Cases 71:35:3 Controls	4.1%	5.4%	0.0%	8.9E-1
Asthma	3:2:0 Cases 71:35:3 Controls	4.1%	5.4%	0.0%	9.5E-1

All P values are from burden analyses. All analyses for quantitative traits were performed using linear mixed models with age and gender as fixed effects, and family ID as a random effect. Clinical outcomes were analyzed using logistic regression with age and gender as covariates. For clinical outcomes, case and control counts are listed as HomRR:HetRA:HomAA. *- Multiple testing correction P value cutoff = 1.1E-3. Abbreviations: ALT, alanine aminotransferase; AST, aspartate aminotransferase; BP, blood pressure; bpm, beats per minute; BMI, body mass index; cm, centimeter; dl, deciliter; eGFR, estimated glomerular filtration rate; g, grams; fl, femtoliter; HDL-C, high density lipoprotein cholesterol; HbA1c, hemoglobin A1C; kg, kilogram; L, liters; LDL-C, low density lipoprotein cholesterol; m^2, meter-squared; MCH, mean corpuscular hemoglobin; MCHC, mean corpuscular hemoglobin concentration; MCV, mean corpuscular volume; min, minutes; mL, milliliters; mmHg, millimeters mercury; pg, picograms; WHR, waist-to-hip ratio; U, units.

### CD33 PheWAS

To determine whether loss of CD33 was associated with any of the phenotypes assessed as part of the broader PGR cohort, a burden phenome-wide association study (PheWAS) was performed on quantitative (Table 4) and binary traits (Table 5). No associations were observed that were significant after correcting for multiple testing. CD33 loss of function was only nominally association with a lower height (β = -0.2, P = 9.4E-4) and parathyroid hormone levels (β = -0.48, P = 8E-3) and higher BMI (β = 0.17, P = 1.34E-2) in the additive model. In the recessive model a nominal association with increasing glucose was observed (β = 0.88, P =4.5E-2)

**Table 4:** Results of a PGR burden PheWAS of quantitative traits using pooled high confidence pLOF variants.

Phenotype	Genotype counts	Additive Model			Recessive Model		
		P value	Beta	SE	P value	Beta	SE
Height	62475:191:3	9.9E-4	-0.21	0.063	5.1E-1	-0.42	0.63
Weight	63949:196:4	4.6E-1	0.048	0.065	4.1E-1	-0.55	0.66
WHR	60135:193:4	7.2E-1	0.024	0.067	3.9E-1	-0.58	0.68
BP diastolic	83863:246:6	1.00	0	0.058	6.8E-1	-0.20	0.47
BP systolic	83841:242:7	7.9E-1	-0.016	0.059	4.3E-1	-0.34	0.43
BMI	61804:188:3	1.3E-2	0.17	0.068	5.5E-1	-0.40	0.68
ASTL	32096:99:2	7.8E-2	0.17	0.094	2.1E-1	1.22	0.97
ALTL	31648:95:2	1.5E-1	0.14	0.095	7.4E-1	0.32	0.96
QT interval	42149:117:4	7.3E-1	0.033	0.096	6.8E-1	0.20	0.48
Parathyroid hormone	7345:26:1	8.0E-3	-0.48	0.18	3.4E-1	-0.94	0.98
Glucose	88562:269:6	5.4E-2	0.11	0.057	4.5E-2	0.88	0.44
Cystatin C	21527:50:1	7.4E-1	-0.044	0.13	6.4E-1	-0.45	0.96
Total Cholesterol	96081:284:6	4.7E-1	0.041	0.056	3.5E-1	0.41	0.44
Triglycerides	95824:284:6	7.8E-1	0.016	0.055	4.4E-1	0.34	0.43
HDL	95779:285:6	6.8E-1	0.023	0.055	8.4E-1	0.088	0.43
LDL	87772:260:6	3.9E-1	0.050	0.058	6.1E-1	0.23	0.44
VLDL (Calculated)	95309:283:6	8.5E-1	0.011	0.055	3.5E-1	0.41	0.43
Creatinine	77858:233:3	3.1E-1	-0.058	0.057	8.6E-1	0.11	0.64
eGFR	74643:223:3	9.7E-1	0.0020	0.061	9.9E-1	-0.0078	0.66

Abbreviations: ALT, alanine amino transferase; AST, aspartate aminotransferase; BP, blood pressure; BMI, body mass index; eGFR, estimated glomerular filtration rate; LDL, low density lipoprotein cholesterol; HDL, high density lipoprotein cholesterol; VLDL, very low density lipoprotein cholesterol.

**Table 5:** Results of a PGR burden PheWAS of binary traits using pooled high confidence pLOF variants.

Phenotype	Cases	Controls	Additive Model			Recessive Model		
**P value**	**Odds ratio**	**SE**	**P value**	**Odds ratio**	**SE**
ASCVD	39013|109|3	51839|156|4	9.2E-1	0.99	0.13	7.9E-1	1.29	0.98
Myocardial infarction	38028|103|3	50705|152|4	7.8E-1	0.96	0.13	9.4E-1	1.08	0.94
Angina	2790|6|0	51850|157|4	2.8E-1	0.69	0.24	7.6E-1	0.34	2.41
Atrial fibrillation/ Irregular heart beats	9450|38|1	90092|257|6	3.3E-1	1.20	0.23	4.0E-1	3.86	1.92
Hypertension	36114|120|2	62644|172|5	5.1E-1	1.08	0.13	5.9E-1	0.59	1.08
Ischemic stroke	3034|13|0	36621|107|3	1.7E-1	1.68	0.64	-	-	-
Hemorrhagic stroke	1876|9|0	37555|108|3	3.6E-1	1.48	0.63	-	-	-
Stroke	6428|24|0	51770|156|4	4.5E-1	1.25	0.36	8.9E-1	0.36	8.86
Type 2 diabetes	29841|100|1	54717|156|4	9.1E-2	1.26	0.17	8.3E-1	0.78	1.41

Abbreviations: ASCVD, atherosclerotic cardiovascular risk.

Given the modest blood cell phenotype observed from the PBMCs collected from the PGR recall study along with previous publications indicating that CD33 may play a role in susceptibility to Alzheimer’s disease and isolated leukemias, a subset of 31 continuous phenotypes and 13 binary outcomes related to hematological clinical endpoints as well as neurological disease were evaluated for association with CD33 LOF in the large whole exome dataset available through UK Biobank. The full list of 27 pLOF variants surveyed as part of the UKBB PheWAS is described in Table 6.

**Table 6:** List of high-confidence pLOF variants identified in UK Biobank used for PheWAS.

Chr:Pos:Ref:Alt (GRCh38)	Consequence	HGVSp	N	AC	Heterozygous Carriers	Homozygous Carriers	Frequency
19:51225139:GC:G	Frameshift variant	p.Leu9CysfsTer21	391427	2	2	0	2.55E-06
19:51225246:G:A	Stop gained	p.Trp22Ter	391427	1	1	0	1.28E-06
19:51225351:T:TG	Frameshift variant	p.Tyr59LeufsTer40	391427	1	1	0	1.28E-06
19:51225352:GGT:G	Frameshift variant	p.Gly58ValfsTer40	391427	2	2	0	2.55E-06
19:51225356:ACT:A	Stop gained	p.Tyr59Ter	391427	2	2	0	2.55E-06
19:51225390:CT:C	Frameshift variant	p.Ser71LeufsTer8	391427	67	67	0	8.56E-05
19:51225409:A:T	Stop gained	p.Lys77Ter	391426	1	1	0	1.28E-06
19:51225445:A:T	Stop gained	p.Arg89Ter	391426	45	45	0	5.75E-05
19:51225493:AT:A	Frameshift variant	p.Ile105ThrfsTer2	391427	1	1	0	1.28E-06
19:51225541:G:T	Stop gained	p.Glu121Ter	391425	3	3	0	3.83E-06
19:51225559:TAC:T	Frameshift variant	p.Tyr127Ter	391423	1	1	0	1.28E-06
19:51225812:A:AGG	Frameshift variant	p.His143GlnfsTer12	391425	1	1	0	1.28E-06
19:51225836:G:GCATTCTAGAACCCGGC	Frameshift variant	p.Thr152IlefsTer24	391426	1	1	0	1.28E-06
19:51225847:CCCGG:C	Frameshift variant	p.Gly156ThrfsTer5	391419	23631	22885	373	0.030
19:51225951:CA:C	Frameshift variant	p.Arg190GlyfsTer9	391426	1	1	0	1.28E-06
19:51225968:C:A	Stop gained	p.Ser195Ter	391427	1	1	0	1.28E-06
19:51225982:AC:A	Frameshift variant	p.Pro201HisfsTer10	391426	1	1	0	1.28E-06
19:51226021:CA:C	Frameshift variant	p.Gln213ArgfsTer2	391427	1	1	0	1.28E-06
19:51226308:G:C	Splice acceptor variant		391423	2	2	0	2.55E-06
19:51226321:ACCCAACAACTGGTATCTTT:A	Frameshift variant	p.Pro238GlnfsTer38	391325	105	101	2	0.00013
19:51235162:CAA:C	Frameshift variant	p.Gln251ArgfsTer47	391197	1	1	0	1.28E-06
19:51235165:G:T	Stop gained	p.Glu252Ter	391411	1	1	0	1.28E-06
19:51235255:T:C	Splice donor variant		391413	1	1	0	1.28E-06
19:51235255:T:G	Splice donor variant		391413	2	2	0	2.55E-06
19:51235677:G:C	Splice donor variant		391424	1	1	0	1.28E-06
19:51235678:T:C	Splice donor variant		391410	499	499	0	0.00064
19:51239662:G:T	Stop gained	p.Glu357Ter	391426	77	77	0	9.84E-05

Abbreviations: Chr, chromosome; pos, position; Ref, reference allele; Alt, alternate allele; and HGVSp, Human Genome Variation Society protein level change. Predicted coding consequence is relative to CD33 isoform 1, NP_001763.3.

Among the quantitative hematological traits queried, platelet crit (β = -0.027, P = 4.0E-6), leukocyte counts (β = -0.027, P = 2.0E-5), and lymphocyte counts (β = -0.024, P = 1.0E-4) showed small but significantly lower levels in CD33 pLOF carriers after Bonferroni multiple testing correction (Table 7; additive model). Nominally significantly lower counts of platelets (β = -0.018, P = 2.1E-3), monocytes (β = -0.018, P = 2.5E-3), and neutrophils (β =-0.019, P = 2.7E-3) as well as platelet/thrombocyte volume (β = -0.012, P = 2.9E-2) were also observed. In contrast, reticulocyte percentage (β =0.025, P = 6.3E-5), total reticulocyte count (β = 0.023, P = 1.8E-4), and high light scatter reticulocyte percentage (β = 0.023, P = 3.1E-4) were significantly higher among pLOF carriers.

**Table 7:** Summary of PheWAS findings for high confidence pLOF variants from the UK Biobank.

Phenotype	UKBB Field(s)	Diagnosis ICD10(s)	N	Beta (additive)	OR (additive)	P value (additive)	Beta (recessive)	OR (recessive)	P value (recessive)
White blood cell (leukocyte) count	30000	NA	379879	-0.027	NA	2.0E-5	-0.10	NA	3.6E-2
Red blood cell (erythrocyte) count	30010	NA	379884	-0.0040	NA	4.6E-1	-0.0080	NA	8.5E-1
Hemoglobin concentration	30020	NA	379883	0.0020	NA	7.0E-1	0.044	NA	2.6E-1
Hematocrit %	30030	NA	379884	-0.0020	NA	7.5E-1	0.012	NA	7.7E-1
Mean corpuscular volume	30040	NA	379882	0.0070	NA	2.7E-2	0.034	NA	4.7E-1
Mean sphered cell volume	30270	NA	373354	-0.0050	NA	4.3E-1	0.029	NA	5.5E-1
Mean corpuscular hemoglobin	30050	NA	379881	0.010	NA	8.1E-2	0.074	NA	1.1E-1
Mean corpuscular hemoglobin concentration	30060	NA	379877	0.011	NA	8.8E-2	0.080	NA	1.1E-1
Red blood cell (erythrocyte) distribution width	30070	NA	379882	-0.0090	NA	1.4E-1	-0.056	NA	2.5E-1
Platelet count	30080	NA	379883	-0.018	NA	2.1E-3	-0.036	NA	4.4E-1
Platelet crit	30090	NA	379880	-0.027	NA	4.0E-6	-0.051	NA	2.7E-1
Mean platelet thrombocyte volume	30100	NA	379879	-0.012	NA	2.9E-2	0.001	NA	9.7E-1
Platelet distribution width	30110	NA	379879	0.0020	NA	7.3E-1	-0.010	NA	8.3E-1
Lymphocyte count	30120	NA	379187	-0.024	NA	1.0E-4	-0.10	NA	3.7E-2
Monocyte count	30130	NA	379187	-0.018	NA	2.5E-3	-0.071	NA	1.3E-1
Neutrophil count	30140	NA	379187	-0.019	NA	2.7E-3	-0.077	NA	1.2E-1
Eosinophil count	30150	NA	379187	-0.012	NA	5.2E-2	-0.12	NA	1.2E-2
Basophil count	30160	NA	379187	-0.0070	NA	2.2E-1	-0.014	NA	7.7E-1
Nucleated red blood cell count	30170	NA	379179	0.000	NA	9.0E-1	0.018	NA	2.1E-1
Lymphocyte %	30180	NA	379192	-0.0030	NA	5.8E-1	-0.019	NA	7.0E-1
Monocyte %	30190	NA	379192	0.0020	NA	7.5E-1	0.0060	NA	9.0E-1
Neutrophil %	30200	NA	379192	0.0040	NA	4.9E-1	0.024	NA	6.3E-1
Eosinophil %	30210	NA	379192	-0.0040	NA	5.7E-1	-0.047	NA	2.4E-1
Basophil %	30220	NA	379192	0.0020	NA	8.0E-1	0.040	NA	4.5E-1
Nucleated red blood cell %	30230	NA	379176	0.000	NA	8.7E-1	0.018	NA	2.0E-1
Reticulocyte %	30240	NA	373353	0.025	NA	6.3E-5	0.053	NA	2.9E-1
Reticulocyte count	30250	NA	373354	0.023	NA	1.8E-4	0.050	NA	3.1E-1
Mean reticulocyte volume	30260	NA	373352	0.0020	NA	7.7E-1	0.048	NA	3.3E-1
Immature reticulocyte fraction	30280	NA	373352	0.0070	NA	2.8E-1	0.0040	NA	9.4E-1
High light scatter reticulocyte %	30290	NA	373354	0.023	NA	3.1E-4	0.049	NA	3.2E-1
High light scatter reticulocyte count	30300	NA	373354	0.021	NA	6.3E-4	0.047	NA	3.4E-1
Monocytic leukemia	41270	C93	391427	0.39	1.48	3.0E-1	-1.01	0.36	6.8E-1
Myeloid leukemia	41270	C92	391427	-0.043	0.96	7.5E-1	-1.01	0.37	3.9E-1
Any leukemia	41270	C91; C92; C93; C94; C95	391427	-0.018	0.98	8.9E-1	-1.01	0.36	3.4E-1
Dementia in Alzheimer’s	130836	F00	391427	-0.14	0.87	8.8E-2	-0.21	0.81	7.5E-1
Vascular dementia	130839	F01	391427	-0.21	0.81	4.4E-2	-0.46	0.63	5.5E-1
Dementia in other diseases	130840	F02	391427	-0.20	0.82	1.4E-1	1.74	5.70	1.4E-1
Unspecified dementia	130842	F03	391427	-0.059	0.94	3.6E-1	0.98	2.66	5.3E-2
Alzheimer’s	131036	G30	391427	-0.091	0.91	2.0E-1	-0.061	0.94	9.1E-1
Any dementia or Alzheimer’s	130836; 130839; 130840; 130842; 131036	F00; F01; F02; F03; G30	391427	-0.094	0.91	5.4E-2	0.59	1.79	1.3E-1
Stroke	131368	I64	391427	0.031	1.03	5.0E-1	0.23	1.26	5.1E-1
Senile cataracts	131164	H25	391427	0.032	1.03	2.0E-1	0.37	1.45	7.7E-2
Other cataracts	131167	H26	391427	-0.025	0.98	2.6E-1	0.14	1.15	4.4E-1
Any cataracts	131164; 131167	H25; H26	391427	0.0040	1.00	8.6E-1	0.33	1.39	2.5E-2

No binary outcomes in UKBB were found to be statistically significant after correction for multiple testing (Table 7). Nominally significant associations found were with a lower vascular dementia risk (OR = 0.80, P = 4E-2; additive model) and a higher rate of cataracts (OR = 1.39, P = 2.5; recessive model). No significant reduction in Alzheimer’s disease risk was observed. There was also no significant association observed between CD33 LOF and myeloid or monocytic leukemias. For these latter phenotypes, the low total number of cases in UKBB may limit the discovery power to detect a signal.

## Discussion

To our knowledge, no systematic evaluation has been reported that ascertains whether loss of CD33 is a modulator of disease risk in humans. Rare individuals with very low or absent CD33 expression and concurrent pathology have been reported. One report describes an infant with low levels of surface CD33 expression, copper deficiency, and myelodysplastic syndrome (10). It is unclear if the pathologies were directly related to CD33 and if the loss of CD33 expression was due to the dysmyelopoiesis present in myelodysplastic syndrome or germline CD33 variants carried by the infant as no DNA sequencing was reported. A second report describes a 68-year-old woman with acute myeloid leukemia and no detectable levels of surface CD33 protein [18]. DNA analysis of a bone marrow aspirate of the patient with active disease, and prior to a bone marrow transplant, revealed a homozygous CD33 deletion (19:51225847:CCCGG:C) that would yield the G156TfsTer5 variant. But without sequencing of a separate non-transformed tissue DNA source, it is unclear if this deletion was a germline variant or a variant present in leukemic cells. To better understand the role of CD33 in human health and disease, we systematically evaluated the impact of germline *CD33* LOF variants in humans through a combination of biochemical characterization of LOF variants, recall of confirmed LOF variant carriers for in-depth phenotyping including profiling of PBMCs, and PheWAS of two large whole exome biobanks (PGR and UKBB).

Within the Pakistani population, CD33 LOF allele frequency was observed to be rare (0.4%) and was not associated with any overt disease states in the PGR biobank after multiple testing corrections. RBG studies additionally showed no obvious enrichment or depletion of disease in families with heterozygous and homozygous LOF variant carriers. Complete blood cell counts using an automated hematology analyzer also showed no significant changes in circulating cells. FACS-based analysis of PBMCs from consenting participants, however, did show increases in CD14^+^ monocytes and a decrease in CD3^+^ T cells in homozygous LOF carriers. It is unclear if the differences observed between the hematology analyzer and FACS analysis are methodologically based (the hematology analyzer does not use the more precise FACS-based cell surface markers for cell population identification) or due to the smaller number of samples from the FACS study. Despite these changed counts, individuals did not report any notable history of recurrent infectious disease. Additionally, the existence of homozygous LOF males in these recall studies who have successfully had children indicate that CD33 is dispensable for male fertility and fetal-derived CD33 is not essential for *in utero* development in males.

In the largely non-Finnish European-based ancestry of UKBB, CD33 pLOF variants occurred at a much higher overall frequency (3% allele frequency) than in Pakistan (0.4% allele frequency). Whether selective forces are behind the nearly 10-fold higher enrichment of CD33 pLOF variants in non-Finnish Europeans compared to South Asians may be worth investigating. Consistent with the data from the PGR dataset and recall studies, CD33 LOF was not associated with any overt disease states—including leukemias—in UKBB. Additionally, like the Pakistani cohort, small yet significant changes in circulating cell compartment profiles were observed. This included reductions in white blood cell counts. The magnitude of these changes is very small. The β = -0.027 for changes in white blood cells in UKBB, for instance, translates to an absolute cell count change of -5.8E4 cells/ml, which is ~0.8% of the mean total WBC count of 6.9E6 cells/ml.

Our results also show the presence of shed CD33 in otherwise healthy individuals. Confirmed LOF variants had significantly lower levels of shed protein and surface expression of CD33 on PBMCs. These results suggest that circulating shed protein may be a sensitive proxy marker for *in situ* protein expression in the absence of access to primary tissue [19].

The absence of notable pathology in both confirmed CD33 LOF and pLOF carriers from PGR and UKBB suggests that CD33 is dispensable for normal human health. A possible limitation of this study, however, is that we analyzed biobanks that did not purposefully recruit a large number of individuals with confirmed immune disease. This could result in an ascertainment bias that would underpower the ability to detect a pathophysiological role of CD33 on the background pre-existing immune dysfunction. Future studies with appropriately constructed case-control designs will be needed to address this issue.

Overall, we observed that CD33 LOF produces small, but significant, perturbations in circulating cells within the myeloid cell lineage. These small changes, however, do not appear to result in any adverse phenotypes. This finding is consistent with the phenotype of the Cd33 knockout mouse, which also shows no serious pathology. Importantly, the absence of a remarkable phenotype associated with life-long CD33 LOF strongly suggests but does not conclusively prove that chronic inhibition/ablation of CD33 as a therapeutic intervention is likely to be safe in humans.

## Methods

### Ethics statement

The Institutional Review Board (IRB) at the Center for Non-Communicable Diseases (IRB: 00007048, IORG0005843, FWAS00014490) approved the study. All participants gave written informed consent.

### In vitro expression studies

*Gene constructs for CD33 wild-type and truncation variants p.G156TFsTer5 and p.P238QFsTer38.* A synthetic gene construct for full-length, reference allele human *CD33*, also called CD33M (RefSeq: NP_001763.3), was codon-optimized for expression in human cells. From the CD33M construct we derived expression vectors for p.G156TFsTer5 (G156TFsTer5) and p.P238QFsTer38 (P238QFsTer38). *CD33* gene constructs were synthesized at GeneWiz in pRS5a expression vector.

#### Cell culture.

Human embryonic kidney HEK293F (293F) cell lines were maintained in suspension culture with free-style 293 expression medium (FS293EM) from Invitrogen in a shaking incubator at 37 °C in the presence of 8% CO2 and 80% humidity with 110 revolutions per minute. Cultures with a viability of >96% were utilized for transfection assays. Transfections were conducted in 100 ml HEK293F cells with 100ug plasmid DNA and 250ug PEI (Polyethylenimine 25K) from Polysciences (Warrington, PA). Cultures were harvested after 48h for analyses.

#### Antibody staining and fluorescence activated cytometry sorting (FACS) of transfected HEK293F cells.

To determine cell surface expression of *CD33* variants in transfected cells, fluorescence-conjugated anti-CD33 mouse monoclonal antibody WM53-PE (ab233577) and mAb HIM3-4 (PE-Cy5) (ab169823) were used from Abcam. WM53-PE and HIM3-4 (PE-Cy5) bind to Ig-V and Ig-C2 domain, respectively (Fig 1B). 48h hours post transfection harvested, cells were washed in DPBS, and resuspended in cold FACS buffer (DPBS supplemented with 1% fetal calf serum and 1% goat serum) at 2.5 million cells/ml. For mouse antibody staining, cell suspensions were blocked for 30min with 1:10 diluted mouse serum prior to staining for 40min with predetermined amount of WM53-PE or HIM3-4 (PE-Cy5). Afterwards, the stained cells were washed in FACS buffer and loaded into 96-well plates for analysis on a Fortessa FACS device.

### MSD binding assays for quantification of serum CD33 proteins

A quantitative assay for measuring serum CD33 levels was developed with recombinant monomeric extracellular domain of CD33M (CD33MECDHis, Fig 1B), a commercial anti-CD33 mAb 3D6 (Ab11031, Abcam), and a recombinant anti-CD33 mAb hSGL3 generated from a sequence previously reported [20]. hSGL3 was used for capturing CD33 and 3D6 for detection with standard bind 96-well MSD plates (MesoScaleDiscovery). Briefly, MSD plates were coated with 1.5ug/ml hSGL3 human IgG1 in phosphate-buffered saline (PBS), pH7.4, at 4 °C overnight. Similar MSD plates were incubated with PBS as negative controls. Plates were blocked with 150ul of 1X KPL Milk Diluent/Blocking Solution (Cat# 5140-0010, SeraCare Life Science) diluted in 1X TBS-Tween 20 (Cat# 28360, Thermo Scientific) on a rocking platform for 60min at room temperature followed by 3 washes with 1X TBS-Tween 20 buffer. A positive standard curve was created with CD33MECDHis in assay buffer (1XTBS-Tween 20 supplemented with 0.2% bovine serum albumin). To test serum samples, 30ul serum was mixed with 270ul assay buffer followed by 2.5-fold serial dilutions. Then 35ul serially diluted CD33MECDHis and serum samples were incubated with hSGL3 coated and uncoated plates for 60min followed by 3 washes. Then the plates were incubated with 10nM 3D6 for 60min. After 3 washes, the plates were further incubated with MSD goat anti-mouse IgG sulfo-tag antibody (R32AC-1, 1:1400 dilution) for 60min. After 3x washing, plates were read with 50ul 1X MSD reading buffer T (R92TC-2). Data were processed in Excel and analyzed in Graph Pad Prism 9.2.0. The statistical significance of CD33 concentrations among three genotype groups was analyzed by one-way ANOVA.

### FACS analysis

**Fig 7:**
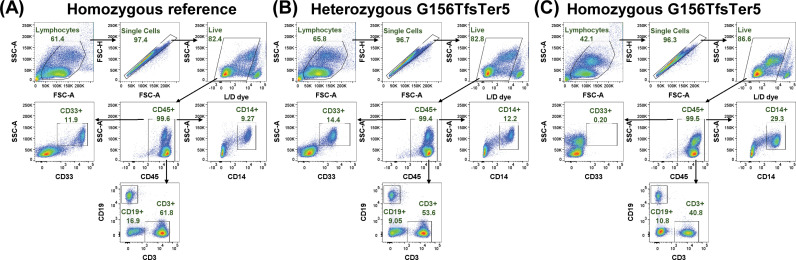
Representative gating strategy data for PBMC FACS studies from a homozygous reference (A), heterozygous p.G156TFTer5 (B), and homozygous p.G156TFsTer5 carriers.

Human PBMCs collected from consenting participants were pre-incubated with Human TruStain FcX (BioLegend Cat. No. 422301) to block Fc-Receptors. Analysis of human CD33 expression levels was performed using the following antibodies: CD45-BUV395 (BD, 563792), CD33-APC (Biolegend, 366626), and Live/Dead Fixable Near IR (Life Technology, L34992). For assessing human PBMC subsets, the following panel was used: CD45-BUV395 (BD, 563792), CD3-BV421 (Biolegend, 317344), CD14-FITC (BD, 561712), CD19-PE (Biolegend, 302208), and Live/Dead Fixable Near IR (Life Technology, L34992). All flow cytometry was performed on a BD Fortessa and analysis was performed using FlowJo V10. The live singlets were first gated by CD45, and the CD45+ population was then further gated by CD33, CD14, CD3 or CD19 to identify the CD33+ myeloid cells, monocytes, T cells or B cells, respectively (see Fig 7 for sample gating strategy).

### Variant QC and annotation

Exome sequencing for the PGR samples was performed at two different locations, at the Broad Institute as described earlier [16] and at the Regeneron Genetics Center. All samples were sequenced at 30X coverage. Samples with low allele balance (< 0.2) or low depth (< 10) were set to missing and variants which had a missing rate > 5% were removed. UKBB exome sequencing was performed by the RGC as previously described [21]. Additional genotype and sample level QC was performed as described previously [22]. Variant annotation was performed using Variant Effect Predictor (VEP) version NNN with the Loftee plugin [23,24]. High confidence pLOF variants were annotated as stop gained, frameshift, splice donor, splice acceptor variants based on Loftee filtering.

#### Exome analysis.

All quantitative traits were transformed by the rank based inverse standard normal function, applied within each genotyping batch. Quantitative traits were analyzed using linear regression as implemented in regenie [25]. All analyses were adjusted for age, sex, age*sex, age ^ 2 and top 10 genetics PCs generated using common genotyping array SNPs. For exomes data, if genotyping array data wasn’t available, PCs were derived from common (MAF > 1%) exome SNPs. Exomes and genomes data were analyzed separately across sequencing centers and meta-analyzed using inverse variance weighted meta-analysis as implemented in METAL [26]. Binary traits were analyzed using logistic regression model, with Firth fallback.

Phenotype definitions: LDL-c, calculated using Friedwald equation, was analyzed by subsetting to individuals who were not on cholesterol lowering drugs, glucose levels were analyzed for participants who were not on oral antidiabetic drugs and creatinine and eGFR (calculated using the CKD-EPI calculation) were subset to participants without heart failure. Myocardial infarction cases were enrolled at time of event as described. Type 2 diabetes was defined as individuals having an HbA1c>= 6.5 or self-reporting to have diabetes or using oral-hypoglycemics. Individuals with a diabetes age of onset less than 30 were excluded from the analysis. Type 2 diabetes controls were ascertained as individuals with HbA1c < 6.5 or no self-reported history of diabetes and their random blood glucose levels were less than 150 mg/dl. Stroke cases, angina, atrial fibrillation/ irregular heartbeats, hypertension were all self-reported. Atherosclerotic cardiovascular disease cases were defined as any individuals with myocardial infarction, stroke, or angina, and controls were healthy individuals without any cardiovascular disease history.

### Recall by genotype study

#### Sanger sequencing.

Whole blood-derived DNA from recalled participants was used to carrier status and zygosity for variants of interest via Sanger sequencing. Sanger sequencing was conducted at Macrogen, Inc (South Korea) or in-house at the CNCD. For Macrogen-processed samples, DNA samples were shipped directly to Macrogen for PCR amplification and Sanger sequencing. PCR primers were designed covering a region of approximately 200 to 300 bases around the variant. For in-house Sanger sequencing, specific primers were designed to amplify the region of interest using Platinum Master Mix (Thermo Scientific, USA). This amplified DNA product was processed using ExoSAP-IT Express PCR Product Cleanup (Thermo Scientific, USA), BigDye XTerminator (Thermo Scientific, USA), then run on Applied Biosystems SeqStudio Genetic Analyzer (Thermo Scientific, USA). Manufacturers’ protocols were followed for all kits.

#### Recall by genotype analyses.

PheWAS for quantitative traits in the recall study was performed using an additive linear mixed model with age and sex as fixed effects, and family ID as a random effect. All quantitative traits were transformed by the rank based inverse standard normal function. Binary traits were analyzed using logistic regression with age and gender as covariates. All analyses were performed as burden analyses, with carriers of different CD33 LOF variants grouped together. All analyses were performed using the statsmodels Python module (version 0.13.0).

#### PBMC isolation.

 20 ml of whole blood from consenting participants was collected in a citrate phosphate dextrose solution with EDTA and heparin. An equal volume of Ca^2+^/Mg^2+^-free DPBS buffer supplemented with 2% FBS was added and mixed. The cell mixture was carefully layered over sterile Ficoll-Paque density 1.077 media (GE Healthcare cat #17-5442-02) and centrifuged at 400xg for 30 min. Mononuclear cells were isolated and washed in Ca^2+^/Mg^2+^-free DPBS buffer supplemented with 2% FBS. After washing, cells were resuspended in wash buffer to a concentration of ~2e7 cells/ml. An equal volume of cell freezing medium (Cryostore CS10, Stemcell Technologies cat 100-1061) was added and cells were slow froze at -80C in a Mr. Frosty Cell freezer (Thermo Scientific cat no 5100-0001). For long-term storage, cells were transferred to liquid nitrogen.

### UK Biobank (UKBB) dataset analyses

UK Biobank analysis was performed in a similar manner to the PGR PheWAS analysis. Data was accessed under approved UK Biobank project 59456. Analyses were restricted to a set of unrelated individuals with high quality genetic data (UKBB Data Field 22020). A quality-controlled set of genotyping array data was used in regenie’s whole genome regression step 1. All quantitative traits were transformed by the rank based inverse standard normal function. Quantitative traits were analyzed using linear regression as implemented in regenie. All analyses were adjusted for age, sex, age*sex, age^2 and the top 10 genetics PCs generated using common genotyping array SNPs. Binary traits were analyzed using a logistic regression model with Firth fallback. Continuous and binary phenotypes related to hematological clinical endpoints as well as neurological disease were selected for evaluation. For quantitative traits, the first available instance was assessed. Binary outcomes were defined based on the presence or absence of ICD10 codes. The specific ICD10 codes evaluated are listed in Table 6. All analyses were done as part of UK Biobank Resource Application Number 59456.
