# Supplementary material for: Human CD33 deficiency is associated with mild alteration of circulating white blood cell counts
Source: PLoS Genet. 2025 Mar 5;21(3):e1011600. doi: 10.1371/journal.pgen.1011600 (PMC11927914; doi:10.1371/journal.pgen.1011600)
Supplement: Attachment — Submitted filename: 20250123 Replies to Comments to the Authors final.docx [file pgen.1011600.s001.docx]

**Dear Editor and Reviewers,**

**We thank everyone for their review of the manuscript and the thoughtful feedback. We have addressed the comments and amended the manuscript accordingly. We believe that these changes strengthen the submission. Please see below.**

**Many thanks,**

**John Dominy**

**Comments to the Authors:
Please note here if the review is uploaded as an attachment.**

Reviewer #1: Dominy et al. report the characterization of three individuals with homozygous loss of function mutations in the CD33 gene identified through the Pakistan Genome Resource. The authors confirmed that these individuals lack surface and soluble CD33 protein expression. There was a slight increase in their circulating CD14+ monocytes and decrease in their CD3+ T cells compared to controls, and comprehensive health assessment showed higher risk of elevated cholesterol and myocardial infarction in CD33 LOF individuals. Additional investigation into the UK Biobank, which contained a larger number of CD33 LOF individuals, found a small but significant correlation with decreased WBC and platelet counts, and higher reticulocyte counts.

CD33 is a well-established target for immunotherapy for myeloid malignancies, and genetic deletion of CD33 in hematopoietic stem cells is being pursued as a method to improve the specificity of CD33 targeted therapy. While there is no experimental evidence to believe that CD33 loss would cause any detrimental effect on hematopoietic cells, a lingering concern remains that when subject to diverse environmental insults a previously unknown role of CD33 in maintaining health would emerge. The current study is significant in that it shows that homozygous CD33 LOF individuals have no overt health problems detected despite comprehensive evaluation. This provides further evidence that CD33 KO strategies can be pursued safely, particularly for diseases that carry a high mortality rate.

I found the paper was well written, the data was clearly presented, and the conclusions were reasonable.

Minor point: line 285 “reductions in CD14+ monocytes” -> should be corrected to “increases in CD14+ monocytes”

- **We corrected this error.**

Reviewer #2: CD33 is genetically associated to Alzheimer’s disease risk and is being investigated as a drug target for inhibitory drugs. This manuscript examines individuals homozygous for CD33 loss of function to determine the tolerability of this approach, and also does pheWAS analyses for heterozygous individuals to examine the possibility of any associated phenotypes that could predict side effects. No significant concerns are identified, suggesting that CD33 inhibition should be a safe therapeutic strategy. The manuscript is logical and well-written and I have no major concerns. Just a couple of points I suggest improving.

Figure 1 shows that the frameshift variants are indeed translated and secreted in a cell system, and apparently the native function of CD33 is not fully understood, so in principle one cannot simply assume they are total LOF. The authors acknowledge this: “In aggregate, these data indicated that frameshift variant proteins were efficiently produced by cells and, if the frameshift variant transcripts were able to escape nonsense mediated mRNA decay in human carriers, a secreted protein could be generated. Measurement of CD33 protein levels in both sera and cells of human variant carriers was therefore necessary to resolve this,” thus cueing up Figure 4. Yet the ELISA diagram in Figure 4 appears to use two antibodies one of which is to the IgV domain (which is retained in the frameshift variants) but the other of which is to the Ig-C2 domain. The text states “A monoclonal antibody recognizing the common IgV domain of all isoforms was used to capture CD33 protein from sera. Detection was accomplished with a monoclonal antibody, 3D6, which binds to the Ig-C2 domain common to all putative isoforms from the recall study”. This statement is supposed to put us at ease that the lack of signal for homozygous frameshift individuals in Figure 4 is genuinely due to lack of protein, and not just due to lack of the detection epitope on a truncated protein. Yet when I try to line up the diagrams in Figure 1A (which shows the mutation positions), 1B (which shows all the domains), and 4A (which shows the immunogen for the ELISA antibodies), it looks as though the codon 156 frameshift does actually abolish the Ig-C2 domain? I suggest that the authors clarify this. It may be helpful if 1) the positions of the codons where frameshifts occur could be superimposed directly on the diagram of mature protein domains from Figure 1B, rather than only on the intron/exon structure diagram, and 2) an explicit statement could be made that the epitope for the ELISA antibodies are at codons XXX-YYY where presumably XXX and YYY are two numbers each lower than 156.

- **We thank the reviewer for paying close attention to the specificity of the reagents in our study. We revised the text and added data to Figure 5.**
- **In summary:**
  - **The exact epitope of the 3D6 antibody within the Ig-C2 domain was not previously known. When we developed the assays presented in Figure 4, we observed that 3D6 detects G156TfsTer5 recombinant protein poorly. Therefore, we developed a second, orthogonal ELISA, presented in Figure 5, to confirm absence of G156TfsTer5 protein in the sera of variant carriers.**

Refs 13 & 20 provide the prior reports on individuals with CD33 deficiency. Kohla 2020 <https://pmc.ncbi.nlm.nih.gov/articles/PMC7036529/> & Papageorgiou 2020 <https://pmc.ncbi.nlm.nih.gov/articles/PMC7153571/> . The authors should spend a few more sentences helping the reader to understand why these reports have not already answered the question of CD33 KO tolerability. From a quick glance it appears this is because Kohla 2020 simply showed absence of the protein on certain cells without any germline DNA analysis at all, while Papageorgiou 2020 showed that the germline DNA was wild-type, suggesting it was only the cancer cells that lacked CD33 expression. This is very different from examining individuals who are germline homozygous KO for CD33, which apparently had never been done prior to the present manuscript, and it would help to guide the reader to this conclusion.

- **In the revised discussion, we describe the studies highlighted by the reviewer. We contrast the findings in those studies to our direct interrogation of the impact of germline homozygous loss of CD33 function in humans. The revised text:**
  - **“To our knowledge, no systematic evaluation has been reported that ascertains whether loss of CD33 is a modulator of disease risk in humans. Rare individuals with very low or absent CD33 expression and concurrent pathology have been reported. One report describes an infant with low levels of surface CD33 expression, copper deficiency, and myelodysplastic syndrome (13). It is unclear if the pathologies were directly related to CD33 and if the loss of CD33 expression was due to the dysmyelopoiesis present in myelodysplastic syndrome or germline CD33 variants carried by the infant as no DNA sequencing was reported. A second report describes a 68-year-old woman with acute myeloid leukemia and no detectable levels of surface CD33 protein (21). DNA analysis of a bone marrow aspirate of the patient with active disease, and prior to a bone marrow transplant, revealed a homozygous CD33 deletion (19:51225847:CCCGG:C) that would yield the G156TfsTer5 variant. But without sequencing of a separate non-transformed tissue DNA source, it is unclear if this deletion was a germline variant or a variant present in leukemic cells. To better understand the role of CD33 in human health and disease, we systematically evaluated the impact of germline *CD33* LOF variants in humans through a combination of biochemical characterization of LOF variants, recall of confirmed LOF variant carriers for in-depth phenotyping including profiling of PBMCs, and PheWAS of two large whole exome biobanks (PGR and UKBB). “**

Reviewer #3: This study by Dominy and colleagues aims to investigate germline loss of function mutations in the CD33 gene. The authors describe three CD33 frameshift mutations that were identified in several individuals from different Pakistani families. Studies of PB-MNCs of heterozygous and homozygous mutation carriers show decreased CD33 levels and decreased CD14+ monocyte and CD3+ T-cell counts. Further analysis of various phenotypes (including CBC parameters) does, however, not show significant associations with CD33 carrier status. Lastly, CD33 is investigated in the UK Biobank, which reveals several CD33 LOF variants and an association with lower platelet, monocyte, and neutrophil counts.
The manuscript is interesting because it systematically investigates CD33 variants in a larger cohort and describes an association of germline CD33 variants with decreased CD33 levels and mildly decreased monocyte and T-cell counts. However, the authors do not answer how the loss of CD33 leads to lower blood counts. In the conclusion, the authors state that based on their study long-term ablation of CD33 is safe. However, this conclusion is not answered by this study.

- **We thank the reviewer for the constructive feedback. We agree that the association of CD33 LOF with modest blood count changes is interesting. An explanatory mechanistic investigation is beyond the scope of our observational human genetics study, which focuses on the therapeutic implications of life-long loss of CD33 without overt pathology.**

Minor points:
Figure 1D/Line 115-116: There is a discrepancy in the description of Fig. 1D (no CD33M detection in medium) vs the results text (“frameshift variants were indeed able to be secreted and at levels that were markedly higher than observed for wild-type protein”) ◊ this should be clarified in the text that CD33M is not secreted according to Fig. 1D

- **This section has been revised for clarity:**
  - **“Probing of total cell lysates demonstrated expression of reference CD33M as well as the frameshift variants, indicating that cells could support generation of the frameshift variants (Figure 1D). Evaluation of CD33 signal in the media revealed detectable levels of secreted frameshift variants but not CD33M reference allele.”**

Table 2: Additional variant information would be helpful (e.g., MAF global/Non-Finnish Europeans/ South Asians, CADD score, phyloP, ACMG sequence variant interpretation, etc.)

- A**dditional variant information has been added to Table 2:**
  - **gnomAD overall allele frequency**
  - **gnomAD South Asian allele**
  - **frequency**
  - **CADD Score**
  - **ClinVar Classification**

Table 5: Did any of the heterozygous or homozygous CD33 LOF variant carriers have recurrent infections or signs of autoimmunity?

- **Table 5 contains analyses of LOF variant carriers from the PGR biobank. The biobank does not have the relevant phenotypic data to comment on recurrent infection or autoimmune disease from recruited participants. Some of these phenotypes were specifically queried, however, as part of the recall by genotype study and shown in Table 3. There was no meaningful association between CD33 LOF and recurrent chest infection, tuberculosis, allergies, asthma, or rheumatoid arthritis in the recalled individuals.**

Figure 6b: A negative/unstained control should be added to the histogram.

- **These data have been added to Figure 6B.**

- Conclusions: Please discuss the discrepancies of low monocyte and T-cell counts in PB-MNCs but no significant association of CD33 mutation status and CBC abnormalities in the PGR cohort.

- **We have added to this in the discussion as extracted below:**
  - **“Within the Pakistani population, CD33 LOF allele frequency was observed to be rare (0.4%) and was not associated with any overt disease states in the PGR biobank after multiple testing corrections. RBG studies additionally showed no obvious enrichment or depletion of disease in families with heterozygous and homozygous LOF variant carriers. Complete blood cell counts using an automated hematology analyzer also showed no significant changes in circulating cells. FACS-based analysis of PBMCs from consenting participants, however, did show increases in CD14+ monocytes and a decrease in CD3+ T cells in homozygous LOF carriers. It is unclear if the differences observed between the hematology analyzer and FACS analysis are due to the small number of samples from the RBG FACS study or due to methodological bias, as the hematology analyzer does not use FACS-based cell surface markers for cell population identification. Despite these changed counts, individuals did not report any notable history of recurrent infectious disease. Additionally, the existence of homozygous LOF males in these recall studies who have successfully had children indicate that CD33 is dispensable for male fertility and fetal-derived CD33 is not essential for in utero development in males.”**

Please discuss the limitations of this study in the conclusions.

- **We now describe these limitations to the discussion:**
  - **“The absence of notable pathology in both confirmed CD33 LOF and pLOF carriers from PGR and UKBB suggests that CD33 is dispensable for normal human health. A possible limitation of this study, however, is that we analyzed biobanks that did not purposefully recruit a large number of individuals with confirmed immune disease. This could result in an ascertainment bias that would underpower the ability to detect a pathophysiological role of CD33 on the background pre-existing immune dysfunction. Future studies with appropriately constructed case-control designs will be needed to address this issue. Overall, we observed that CD33 LOF produces small but significant perturbations in circulating cells within the myeloid cell lineage. These small changes, however, do not correlate to adverse phenotypes. This finding is consistent with phenotype of the Cd33 knockout mouse, which also shows no serious pathology. Importantly, the absence of a remarkable phenotype associated with life-long CD33 LOF suggests that chronic inhibition/ablation of CD33 as a therapeutic intervention may be safe in humans.”**

It may be easier for a potential reader to stay consistent with numbers such as p values (either decimal numbers or scientific notation).

- **The P values have all been converted to scientific notation throughout the text.**
