# Supplementary material for: Human CD33 deficiency is associated with mild alteration of circulating white blood cell counts
Source: PLoS Genet. 2025 Mar 5;21(3):e1011600. doi: 10.1371/journal.pgen.1011600 (PMC11927914; doi:10.1371/journal.pgen.1011600)
Supplement: Peer review history

# Author comment: Author response to Decision Letter 0

**DOI:** 10.1371/journal.pgen.1011600.r001

9 Sep 2024

# Peer review: Decision Letter 0

**DOI:** 10.1371/journal.pgen.1011600.r002

**Authors:** Anne O'Donnell-Luria

17 Dec 2024

PGENETICS-D-24-01045

Human CD33 deficiency is associated with mild alteration of circulating white blood cell counts

PLOS Genetics

Dear Dr. Dominy,

Thank you for submitting your manuscript to PLOS Genetics. After careful consideration, we feel that it has merit but does not fully meet PLOS Genetics's publication criteria as it currently stands. Therefore, we invite you to submit a revised version of the manuscript that addresses the points raised during the review process.

Please submit your revised manuscript within 30 days Jan 16 2025 11:59PM. If you will need more time than this to complete your revisions, please reply to this message or contact the journal office at plosgenetics@plos.org. When you're ready to submit your revision, log on to https://www.editorialmanager.com/pgenetics/ and select the 'Submissions Needing Revision' folder to locate your manuscript file.

Please include the following items when submitting your revised manuscript:

* A rebuttal letter that responds to each point raised by the editor and reviewer(s). You should upload this letter as a separate file labeled 'Response to Reviewers'. This file does not need to include responses to formatting updates and technical items listed in the 'Journal Requirements' section below.

* A marked-up copy of your manuscript that highlights changes made to the original version. You should upload this as a separate file labeled 'Revised Manuscript with Track Changes'.

* An unmarked version of your revised paper without tracked changes. You should upload this as a separate file labeled 'Manuscript'.

If you would like to make changes to your financial disclosure, competing interests statement, or data availability statement, please make these updates within the submission form at the time of resubmission. Guidelines for resubmitting your figure files are available below the reviewer comments at the end of this letter.

We look forward to receiving your revised manuscript.

Kind regards,

Anne O'Donnell-Luria, MD, PhD

Section Editor

PLOS Genetics

Aimée Dudley

Editor-in-Chief

PLOS Genetics

Anne Goriely

Editor-in-Chief

PLOS Genetics

**Additional Editor Comments:**

Apologies for the longer review process here - we had some trouble identifying reviewers but thankfully did eventually find 3 reviewers who were able to provide thoughtful feedback on your manuscript. I would be happy to consider a revised version of this manuscript.

**Journal Requirements:**

1) We ask that a manuscript source file is provided at Revision. Please upload your manuscript file as a .doc, .docx, .rtf or .tex. If you are providing a .tex file, please upload it under the item type u2018LaTeX Source Fileu2019 and leave your .pdf version as the item type u2018Manuscriptu2019.

2) Please provide an Author Summary. This should appear in your manuscript between the Abstract (if applicable) and the Introduction, and should be 150-200 words long. The aim should be to make your findings accessible to a wide audience that includes both scientists and non-scientists. Sample summaries can be found on our website under Submission Guidelines:

https://journals.plos.org/plosgenetics/s/submission-guidelines#loc-parts-of-a-submission

3) We do not publish any copyright or trademark symbols that usually accompany proprietary names, eg ©,  ®, or TM  (e.g. next to drug or reagent names). Therefore please remove all instances of trademark/copyright symbols throughout the text, including:

- TM on Lines: 355, 395, 396, 397, and 398.

4) Your manuscript is missing the following sections: Discussion.  Please ensure all required sections are present and in the correct order. Make sure section heading levels are clearly indicated in the manuscript text, and limit sub-sections to 3 heading levels. An outline of the required sections can be consulted in our submission guidelines here:

https://journals.plos.org/plosgenetics/s/submission-guidelines#loc-parts-of-a-submission

5) Please upload all main figures as separate Figure files in .tif or .eps format. For more information about how to convert and format your figure files please see our guidelines:

https://journals.plos.org/plosgenetics/s/figures

**Reviewers' comments:**

Reviewer's Responses to Questions

**Comments to the Authors:**

**Please note here if the review is uploaded as an attachment.**

Reviewer #1: Dominy et al. report the characterization of three individuals with homozygous loss of function mutations in the CD33 gene identified through the Pakistan Genome Resource. The authors confirmed that these individuals lack surface and soluble CD33 protein expression. There was a slight increase in their circulating CD14+ monocytes and decrease in their CD3+ T cells compared to controls, and comprehensive health assessment showed higher risk of elevated cholesterol and myocardial infarction in CD33 LOF individuals. Additional investigation into the UK Biobank, which contained a larger number of CD33 LOF individuals, found a small but significant correlation with decreased WBC and platelet counts, and higher reticulocyte counts.

CD33 is a well-established target for immunotherapy for myeloid malignancies, and genetic deletion of CD33 in hematopoietic stem cells is being pursued as a method to improve the specificity of CD33 targeted therapy. While there is no experimental evidence to believe that CD33 loss would cause any detrimental effect on hematopoietic cells, a lingering concern remains that when subject to diverse environmental insults a previously unknown role of CD33 in maintaining health would emerge. The current study is significant in that it shows that homozygous CD33 LOF individuals have no overt health problems detected despite comprehensive evaluation. This provides further evidence that CD33 KO strategies can be pursued safely, particularly for diseases that carry a high mortality rate.

I found the paper was well written, the data was clearly presented, and the conclusions were reasonable.

Minor point: line 285 “reductions in CD14+ monocytes” -> should be corrected to “increases in CD14+ monocytes”

Reviewer #2: CD33 is genetically associated to Alzheimer’s disease risk and is being investigated as a drug target for inhibitory drugs. This manuscript examines individuals homozygous for CD33 loss of function to determine the tolerability of this approach, and also does pheWAS analyses for heterozygous individuals to examine the possibility of any associated phenotypes that could predict side effects. No significant concerns are identified, suggesting that CD33 inhibition should be a safe therapeutic strategy. The manuscript is logical and well-written and I have no major concerns. Just a couple of points I suggest improving.

Figure 1 shows that the frameshift variants are indeed translated and secreted in a cell system, and apparently the native function of CD33 is not fully understood, so in principle one cannot simply assume they are total LOF. The authors acknowledge this: “In aggregate, these data indicated that frameshift variant proteins were efficiently produced by cells and, if the frameshift variant transcripts were able to escape nonsense mediated mRNA decay in human carriers, a secreted protein could be generated. Measurement of CD33 protein levels in both sera and cells of human variant carriers was therefore necessary to resolve this,” thus cueing up Figure 4. Yet the ELISA diagram in Figure 4 appears to use two antibodies one of which is to the IgV domain (which is retained in the frameshift variants) but the other of which is to the Ig-C2 domain. The text states “A monoclonal antibody recognizing the common IgV domain of all isoforms was used to capture CD33 protein from sera. Detection was accomplished with a monoclonal antibody, 3D6, which binds to the Ig-C2 domain common to all putative isoforms from the recall study”. This statement is supposed to put us at ease that the lack of signal for homozygous frameshift individuals in Figure 4 is genuinely due to lack of protein, and not just due to lack of the detection epitope on a truncated protein. Yet when I try to line up the diagrams in Figure 1A (which shows the mutation positions), 1B (which shows all the domains), and 4A (which shows the immunogen for the ELISA antibodies), it looks as though the codon 156 frameshift does actually abolish the Ig-C2 domain? I suggest that the authors clarify this. It may be helpful if 1) the positions of the codons where frameshifts occur could be superimposed directly on the diagram of mature protein domains from Figure 1B, rather than only on the intron/exon structure diagram, and 2) an explicit statement could be made that the epitope for the ELISA antibodies are at codons XXX-YYY where presumably XXX and YYY are two numbers each lower than 156.

Refs 13 & 20 provide the prior reports on individuals with CD33 deficiency. Kohla 2020 https://pmc.ncbi.nlm.nih.gov/articles/PMC7036529/ & Papageorgiou 2020 https://pmc.ncbi.nlm.nih.gov/articles/PMC7153571/ . The authors should spend a few more sentences helping the reader to understand why these reports have not already answered the question of CD33 KO tolerability. From a quick glance it appears this is because Kohla 2020 simply showed absence of the protein on certain cells without any germline DNA analysis at all, while Papageorgiou 2020 showed that the germline DNA was wild-type, suggesting it was only the cancer cells that lacked CD33 expression. This is very different from examining individuals who are germline homozygous KO for CD33, which apparently had never been done prior to the present manuscript, and it would help to guide the reader to this conclusion.

Eric Vallabh Minikel

December 16, 2024

Reviewer #3: This study by Dominy and colleagues aims to investigate germline loss of function mutations in the CD33 gene. The authors describe three CD33 frameshift mutations that were identified in several individuals from different Pakistani families. Studies of PB-MNCs of heterozygous and homozygous mutation carriers show decreased CD33 levels and decreased CD14+ monocyte and CD3+ T-cell counts. Further analysis of various phenotypes (including CBC parameters) does, however, not show significant associations with CD33 carrier status. Lastly, CD33 is investigated in the UK Biobank, which reveals several CD33 LOF variants and an association with lower platelet, monocyte, and neutrophil counts.

The manuscript is interesting because it systematically investigates CD33 variants in a larger cohort and describes an association of germline CD33 variants with decreased CD33 levels and mildly decreased monocyte and T-cell counts. However, the authors do not answer how the loss of CD33 leads to lower blood counts. In the conclusion, the authors state that based on their study long-term ablation of CD33 is safe. However, this conclusion is not answered by this study.

Minor points:

- Figure 1D/Line 115-116: There is a discrepancy in the description of Fig. 1D (no CD33M detection in medium) vs the results text (“frameshift variants were indeed able to be secreted and at levels that were markedly higher than observed for wild-type protein”) � this should be clarified in the text that CD33M is not secreted according to Fig. 1D

- Table 2: Additional variant information would be helpful (e.g., MAF global/Non-Finnish Europeans/ South Asians, CADD score, phyloP, ACMG sequence variant interpretation, etc.)

- Table 5: Did any of the heterozygous or homozygous CD33 LOF variant carriers have recurrent infections or signs of autoimmunity?

- Figure 6b: A negative/unstained control should be added to the histogram.

- Conclusions: Please discuss the discrepancies of low monocyte and T-cell counts in PB-MNCs but no significant association of CD33 mutation status and CBC abnormalities in the PGR cohort.

- Please discuss the limitations of this study in the conclusions.

- It may be easier for a potential reader to stay consistent with numbers such as p values (either decimal numbers or scientific notation).

**********

**Have all data underlying the figures and results presented in the manuscript been provided?**

Large-scale datasets should be made available via a public repository as described in the *PLOS Genetics* data availability policy , and numerical data that underlies graphs or summary statistics should be provided in spreadsheet form as supporting information.

Reviewer #1: Yes

Reviewer #2: Yes

Reviewer #3: Yes

**********

PLOS authors have the option to publish the peer review history of their article (what does this mean? ). If published, this will include your full peer review and any attached files.

If you choose “no”, your identity will remain anonymous but your review may still be made public.

**Do you want your identity to be public for this peer review?** For information about this choice, including consent withdrawal, please see our Privacy Policy .

Reviewer #1: No

Reviewer #2: **Yes: ** Eric Vallabh Minikel

Reviewer #3: No

[NOTE: If reviewer comments were submitted as an attachment file, they will be attached to this email and accessible via the submission site. Please log into your account, locate the manuscript record, and check for the action link "View Attachments". If this link does not appear, there are no attachment files.]

**Figure resubmission:**

While revising your submission, please upload your figure files to the Preflight Analysis and Conversion Engine (PACE) digital diagnostic tool, https://pacev2.apexcovantage.com/. PACE helps ensure that figures meet PLOS requirements. To use PACE, you must first register as a user. Registration is free. Then, login and navigate to the UPLOAD tab, where you will find detailed instructions on how to use the tool. If you encounter any issues or have any questions when using PACE, please email PLOS at figures@plos.org. Please note that Supporting Information files do not need this step. If there are other versions of figure files still present in your submission file inventory at resubmission, please replace them with the PACE-processed versions.

**Reproducibility:**

To enhance the reproducibility of your results, we recommend that authors deposit laboratory protocols in protocols.io, where a protocol can be assigned its own identifier (DOI) such that it can be cited independently in the future. Additionally, PLOS ONE offers an option to publish peer-reviewed clinical study protocols. Read more information on sharing protocols at https://plos.org/protocols?utm_medium=editorial-email&utm_source=authorletters&utm_campaign=protocols

# Author comment: Author response to Decision Letter 1

**DOI:** 10.1371/journal.pgen.1011600.r003

29 Jan 2025

Attachment Submitted filename: 20250123 Replies to Comments to the Authors final.docx

# Editor report: Decision Letter 1

**DOI:** 10.1371/journal.pgen.1011600.r004

**Authors:** Gregory M. Cooper

29 Jan 2025

Dear Dr Dominy,

We are pleased to inform you that your manuscript entitled "Human CD33 deficiency is associated with mild alteration of circulating white blood cell counts" has been editorially accepted for publication in PLOS Genetics. Congratulations!

Before your submission can be formally accepted and sent to production you will need to complete our formatting changes, which you will receive in a follow up email. Please be aware that it may take several days for you to receive this email; during this time no action is required by you. Please note: the accept date on your published article will reflect the date of this provisional acceptance, but your manuscript will not be scheduled for publication until the required changes have been made.

Once your paper is formally accepted, an uncorrected proof of your manuscript will be published online ahead of the final version, unless you’ve already opted out via the online submission form. If, for any reason, you do not want an earlier version of your manuscript published online or are unsure if you have already indicated as such, please let the journal staff know immediately at plosgenetics@plos.org.

In the meantime, please log into Editorial Manager at https://www.editorialmanager.com/pgenetics/, click the "Update My Information" link at the top of the page, and update your user information to ensure an efficient production and billing process. Note that PLOS requires an ORCID iD for all corresponding authors. Therefore, please ensure that you have an ORCID iD and that it is validated in Editorial Manager. To do this, go to ‘Update my Information’ (in the upper left-hand corner of the main menu), and click on the Fetch/Validate link next to the ORCID field.  This will take you to the ORCID site and allow you to create a new iD or authenticate a pre-existing iD in Editorial Manager.

If you have a press-related query, or would like to know about making your underlying data available (as you will be aware, this is required for publication), please see the end of this email. If your institution or institutions have a press office, please notify them about your upcoming article at this point, to enable them to help maximise its impact. Inform journal staff as soon as possible if you are preparing a press release for your article and need a publication date.

Thank you again for supporting open-access publishing; we are looking forward to publishing your work in PLOS Genetics!

Yours sincerely,

Gregory M. Cooper, PhD

Section Editor

PLOS Genetics

Anne O'Donnell-Luria

Section Editor

PLOS Genetics

Aimée Dudley

Editor-in-Chief

PLOS Genetics

Anne Goriely

Editor-in-Chief

PLOS Genetics

www.plosgenetics.org

Twitter: @PLOSGenetics

----------------------------------------------------

Comments from the reviewers (if applicable):

----------------------------------------------------

**Data Deposition**

If you have submitted a Research Article or Front Matter that has associated data that are not suitable for deposition in a subject-specific public repository (such as GenBank or ArrayExpress), one way to make that data available is to deposit it in the Dryad Digital Repository . As you may recall, we ask all authors to agree to make data available; this is one way to achieve that. A full list of recommended repositories can be found on our website .

The following link will take you to the Dryad record for your article, so you won't have to re‐enter its bibliographic information, and can upload your files directly: 

http://datadryad.org/submit?journalID=pgenetics&manu=PGENETICS-D-24-01045R1

More information about depositing data in Dryad is available at http://www.datadryad.org/depositing. If you experience any difficulties in submitting your data, please contact help@datadryad.org for support.

Additionally, please be aware that our data availability policy  requires that all numerical data underlying display items are included with the submission, and you will need to provide this before we can formally accept your manuscript, if not already present.

----------------------------------------------------

**Press Queries**

If you or your institution will be preparing press materials for this manuscript, or if you need to know your paper's publication date for media purposes, please inform the journal staff as soon as possible so that your submission can be scheduled accordingly. Your manuscript will remain under a strict press embargo until the publication date and time. This means an early version of your manuscript will not be published ahead of your final version. PLOS Genetics may also choose to issue a press release for your article. If there's anything the journal should know or you'd like more information, please get in touch via plosgenetics@plos.org .

# Editor report: Acceptance letter

**DOI:** 10.1371/journal.pgen.1011600.r005

**Authors:** Gregory M. Cooper

PGENETICS-D-24-01045R1

Human CD33 deficiency is associated with mild alteration of circulating white blood cell counts

Dear Dr Dominy,

We are pleased to inform you that your manuscript entitled "Human CD33 deficiency is associated with mild alteration of circulating white blood cell counts" has been formally accepted for publication in PLOS Genetics! Your manuscript is now with our production department and you will be notified of the publication date in due course.

The corresponding author will soon be receiving a typeset proof for review, to ensure errors have not been introduced during production. Please review the PDF proof of your manuscript carefully, as this is the last chance to correct any errors. Please note that major changes, or those which affect the scientific understanding of the work, will likely cause delays to the publication date of your manuscript.

Soon after your final files are uploaded, unless you have opted out or your manuscript is a front-matter piece, the early version of your manuscript will be published online. The date of the early version will be your article's publication date. The final article will be published to the same URL, and all versions of the paper will be accessible to readers.

Thank you again for supporting PLOS Genetics and open-access publishing. We are looking forward to publishing your work!

With kind regards,

Anita Estes

PLOS Genetics

On behalf of:

The PLOS Genetics Team

Carlyle House, Carlyle Road, Cambridge CB4 3DN | United Kingdom

plosgenetics@plos.org | +44 (0) 1223-442823

plosgenetics.org | Twitter: @PLOSGenetics
